# Potential Therapeutic Mechanism of Radix Angelicae Biseratae and Dipsaci Radix Herb Pair against Osteoarthritis: Based on Network Pharmacology and Molecular Docking

**DOI:** 10.1155/2023/2140327

**Published:** 2023-04-14

**Authors:** Yujiang Xi, Ting Zhao, Mingqin Shi, Xiaoyu Zhang, Yanyuan Bao, Jiamei Gao, Jiayan Shen, Hui Wang, Zhaohu Xie, Qi Wang, Zhaofu Li, Dongdong Qin

**Affiliations:** ^1^The First School of Clinical Medicine, Yunnan University of Chinese Medicine, Kunming, China; ^2^School of Basic Medical Sciences, Yunnan University of Chinese Medicine, Kunming, China

## Abstract

**Background:**

A major contributor to older disability is osteoarthritis. Radix Angelicae Biseratae (known as Duhuo in China, DH, the dried rhizome of Angelica pubescens) and Dipsaci Radix (known as Xuduan in China, XD, the dried rhizome of Dipsacus asper Wall) herb pair (DXHP) is widely used to treat osteoarthritis, but the underlying molecular mechanisms still have not been revealed. This research aimed to illustrate the therapeutic mechanism of DXHP against osteoarthritis through the techniques of network pharmacology and molecular docking.

**Methods:**

Gene targets for osteoarthritis and active ingredients for DXHP were screened based on the pharmacology public database and the gene-disease target database. The software program Cytoscape was used to visualize the active chemical target-disease gene network. The STRING biological information website was used to investigate protein interactions. On the Metascape bioinformatics website, Gene Ontology (GO) and Kyoto Encyclopedia of Genes and Genomes (KEGG) pathway enrichment were carried out. The molecular docking of the important chemicals and primary targets identified by the aforementioned screening was performed using Autodock software.

**Results:**

Twenty-six active substances from the DXHP that had strong connections to 138 osteoarthritis-related targets were screened out. According to network analysis, TNF, GAPDH, IL-6, AKT-1, IL-1B, and VEGFA are prospective therapeutic targets, while osthole, cauloside A, ammidin, angelicone, beta-sitosterol, and asperosaponin VI may be significant active components. 1705 biological processes (BP), 155 molecular functions (MF), and 89 cellular components (CC) were identified by GO analysis. KEGG analysis indicated that IL-17, NF-kappa B, HIF-1, MAPK, and AGE-RAGE signaling pathways are potentially involved. Molecular docking showed that cauloside A, osthole, and *β*-sitosterol have excellent binding activity with main targets.

**Conclusions:**

This study comprehensively illuminated the active ingredients, potential targets, primary pharmacological effects, and relevant mechanisms of the DXHP in the treatment of OA. These findings provide fresh thoughts into the therapeutic mechanisms of the main active ingredients of DXHP and provide a reference for further exploration and clinical applications of DXHP.

## 1. Introduction

Destruction of cartilage, remodeling of the subchondral bone, the development of osteophytes, and synovial inflammation are all symptoms of osteoarthritis (OA), a degenerative joint disease [1–3]. OA is directly associated to heredity, age, obesity, injury, and chronic inflammation [4–7]. As populations of aging and obese individuals have grown in size, the prevalence of OA has also increased and is now considered a major public health problem worldwide [8]. According to recent studies, the global prevalence of OA grew by 48% between 1990 and 2019 [3, 9], and that OA affects more than 500 million persons, occurring most commonly in postmenopausal women over age 50 [10]. Some guidelines indicate that nonsteroidal anti-inflammatory drugs (NSAIDs) are the most crucial medications for OA [11, 12]. This class of drugs achieves its anti-inflammatory and analgesic effects by inhibiting cyclooxygenase (COX), a key enzyme in the metabolism of arachidonic acid (AA) [13]. However, the use of COX inhibitors alone is not ideal. Studies have shown that AA can also be metabolized by lipoxygenase (LOX) to produce inflammatory substances such as leukotrienes [14]. Therefore, single introduction of COX inhibitors cannot limit production of inflammatory mediators but instead stimulates and increases the release of inflammatory mediators in the LOX pathway, resulting in adverse reactions [15]. The current mainstream treatment options for OA have limited effects on delaying the development of joint inflammation. In the long term, such drugs cause serious damage to the functions of the digestive tract, liver, and kidneys [16, 17]. In addition, long-term use accelerates the progression of arthritis [18]. Therefore, research into alternative therapies that can contribute to the development of new drugs could improve treatment options for OA.

Traditional Chinese medicine (TCM), as a supplementary therapy, has accumulated rich theoretical knowledge that can potentially help advance clinical prevention and treatment strategies for OA [19]. The earliest research on OA in TCM can be traced back thousands of years to the ancient text “Huangdi Neijing [20].” In recent years, TCM has been suggested to have anti-inflammatory, anti-apoptotic, anti-oxidative, anti-metabolic, and proliferative effects in the treatment of OA diseases [21]. Herb pair refers to the combination of drugs that can be used simultaneously in TCM clinical to enhance efficacy or reduce adverse reactions. Compared with TCM formulas, herb pairs have a more defined synergistic mechanism [22]. *Radix Angelicae Biseratae* (known as Duhuo (DH), the dried rhizome of *Angelica pubescens*) and *Dipsaci Radix* (known as Xuduan (XD), the dried rhizome of *Dipsacus asper* Wall) are a classic herbal drug combination that has been widely used in TCM. Clinical statistical studies have shown that DXHP is one of the most frequently used Chinese medicine pairs in the treatment of OA [23]. In modern pharmacological research, the active ingredients in DH are linked to antioxidant, anti-inflammatory, and analgesic effects [24], while the active components of XD purportedly protect against bone loss, promote cartilage formation, and improve bone metabolism [25]. In addition, the pharmacological compatibility mechanism of DXHP is similarly reflected in the TCM theory. DXHP originated from Duhuo Xuduan decoction in the ancient Chinese medical book “Waitai Miyao.” Chinese Medicine Pharmacopoeia points out that DH helps relieve symptoms of rheumatism, particularly symptoms associated with dampness, swelling, and pain. XD supposedly tonifies the liver and kidneys, nourishes the muscles and bones, and repair fracture [26]. Hence, it is highly essential to research the pharmacological mechanism of DXHP therapy as a possible means of improving the treatment of OA. However, the molecular mechanism of DXHP in the treatment of OA remains unclear.

**Figure 1:**
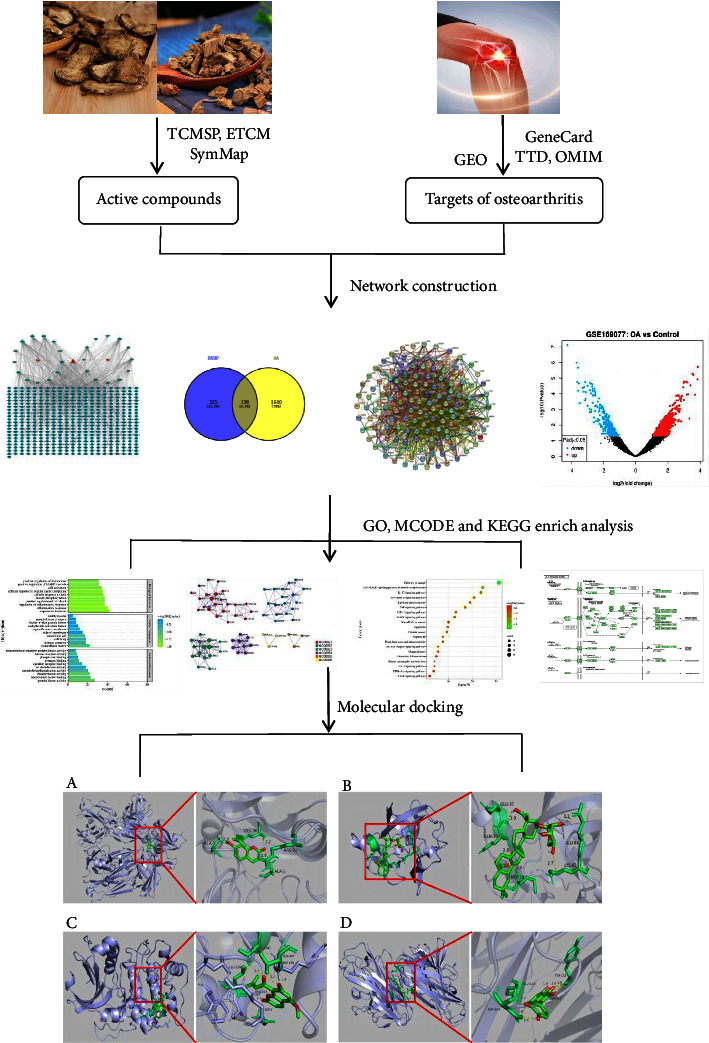
The flowchart of network pharmacology-based prediction and molecular docking technology.

In recent years, network pharmacology, which combines multidisciplinary knowledge and methods, has the characteristics of being systematic and holistic. Molecular docking can calculate the binding energies between ligands and receptors and predict reasonable binding mode and has been used to explore the molecular process of drug active ingredients acting on the human body. Therefore, the aim of this research is to clarify the molecular underpinnings behind the effects of DXHP on OA using network pharmacology and molecular docking technologies. The specific technical route adopted in this study is shown in Figure 1.

## 2. Materials and Methods

### 2.1. Acquisition of Bioactive Compounds of DXHP

To obtain more comprehensive compositional data, the active ingredients of DH and XD were retrieved from three herbal pharmacological databases, including the TCMSP data platform (https://tcmsp-e.com/) [27], the ETCM data platform (https://www.tcmip.cn/ETCM/index.html) [28], and the SymMap data platform (https://www.symmap.org/) [29]. According to previous studies and recommended standards [30], oral bioavailability (OB) ≥30% is regarded as having an excellent absorption. As the selection criteria for “drug-like” compounds in traditional Chinese medicinal materials, DL ≥0.18 is thought to be appropriate for drug design. Therefore, OB and DL criteria were chosen to investigate compounds that meet basic pharmacokinetic criteria for effective drug development [31]. Finally, we searched PubMed and CNKI databases for compounds that did not meet the criteria but had been proved to have significant therapeutic effects on OA.

### 2.2. Target Prediction for Compounds

The active ingredients obtained after the aforementioned screening were queried by PubChem (database https://pubchem.ncbi.nlm.nih.gov/) for SMILES codes of their potential ingredients, which were entered into Swiss Target Prediction (https://www.swisstargetprediction.ch/) for further prediction of active ingredient targets, setting the screening parameter criteria to the probability value >0.1. To normalize gene nomenclature and species and avoid over-annotation of homologous proteins, these target genes were transformed into matching gene symbols that corresponded to the “*Homo sapiens*” species using the UniProt information system (https://www.uniprot.org/) [32].

### 2.3. Collecting Herb-Disease Genes

We retrieved OA-related genes from the GEO data platform (https://www.ncbi.nlm.nih.gov/geo/) [33] database using “osteoarthritis” as a keyword search. The datasets were processed using the robust multiarray average algorithm for background correction and matrix data normalization. The screening conditions for significantly differential genes were *P* < 0.05 and |log FC| >1.5. The differentially expressed genes for OA were obtained. Then, we search the OA-related genes in the following databases: GeneCards (https://www.genecards.org) [34], the TTD information system (https://db.idrblab.net/ttd) [35], and the OMIM information system (https://www.omim.org) [36]. For these searches, the species source was limited to “*Homo sapiens*.” The above-obtained genes were merged, and the duplicated genes were deleted to obtain the defined disease targets of osteoarthritis. Subsequently, the OA-related targets were matched with the active compound targets of DXHP to obtain the target of DXHP in the treatment of OA. The Venn diagram was drawn using the Venny2.1 plotter (https://bioinfogp.cnb.csic.es/tools/venny/index.html).

### 2.4. Protein-Protein Interaction (PPI) Network

Based on the STRING Bioinformatics system (https://www.string-db.org/), a protein-protein interaction (PPI) network was crafted for the common targets of compounds and diseases [37]. The choice of species was human, and the confidence level was set to >0.4. Others parameters keep the default settings. To identify the primary target genes for OA therapy that have a strong correlation with DXHP, the topological properties of common targets were analyzed using the CytoNCA topology analyzer (A plugin for Cytoscape). The network analyzer tool was applied for topology analysis, referring to three parameters, namely the degree centrality value (DC), the betweenness centrality value (BC), and the closeness centrality value (CC). The greater the DC of the node, the higher the importance of the node in a PPI network. Targets with all three parameters above average were selected and ordered by the degree centrality value.

### 2.5. Protein-Protein Interaction Enrichment MCODE Analysis

MCODE, as a clustering algorithm, helps to capture modules with high-quality biological processes in a large PPI spherical network and helps to discover a subset of targets that are closely related to that functional module. MCODE subcluster enrichment analysis was performed on the PPI network. PPI enrichment analysis used the following databases: STRING, InWebIM, OmniPath, and BioGRID. Only the STRING Bioinformatics system (Physical Score >0.132) and BioGRID data are used in the Physical Interaction module. A subset of the proteins in the resultant network physically interacts with at least one other item on the list. The MCODE methodology was used to find components of highly linked networks in subnetworks with 3 to 500 proteins.

### 2.6. Functional Annotation from Gene Ontology (GO) and Pathway Enrichment Analysis from the Kyoto Encyclopedia of Genes and Genomes (KEGG)

The Metascape database (https://metascape.org/) is a powerful broad-coverage and fast-updating gene function annotation analysis tool that can analyze a large number of gene or protein functions. In order to effectively research the biological ontology of DXHP in the regulation of OA and to clarify the biological process of each core target protein and its function in signaling pathway transduction, GO functional annotation and KEGG pathway enrichment analysis were performed based on the Metascape biological system. The common targets of DXHP and OA obtained in the above screening were imported into the target gene list, with confined to just human species and correction of all target genes to their recognized gene symbols. Multiple comparisons were performed using *Benjamini–Hochberg's* FDR correction to avoid false positives. *Bonferroni-correctedP* values <0.01 for GO and KEGG terms were considered significant. The minimal count for the KEGG analysis was 3, and the enrichment factor was more than 1.5. Finally, the top 20 items were picked, and the annotated chart was formed on the Bioinformatics (https://www.bioinformatics.com/) platform for visualization.

### 2.7. Molecular Docking Verification of Compound Target

In network pharmacology, molecular docking is a critical tool for verifying compound-target interactions. It works by combining proteins of known targets obtained from network pharmacology with small compounds of active ingredients in natural drugs and then evaluating the strength and activity of the binding [38]. The mol2 format files of the main active components of DXHP were searched and obtained from the PubChem database in advance. Next, the selected protein's best resolution 3D structure was acquired in the PDB database (https://www.rcsb.org/) and their PDBID files were downloaded. For these 3D structures, the species source was limited to “*Homo sapiens*” and contains the crystal structure of the complete pocket. MGLtool 1.5.7 was used to process the protein by adding hydrogen, calculating the charge, merging the nonpolar hydrogen, and saved as a PDBQT format file as a docking ligand. Gridbox coordinates and docking box sizes were set, and molecular docking was performed using Autodock Vina1.1.2. The lowest binding energy score conformation was selected, with lower binding energy scores indicating better docking activity and strength. The molecular docking structures were demonstrated using Pymol 2.3.

## 3. Results

### 3.1. Screening of DXHP Active Compounds

**Figure 2:**
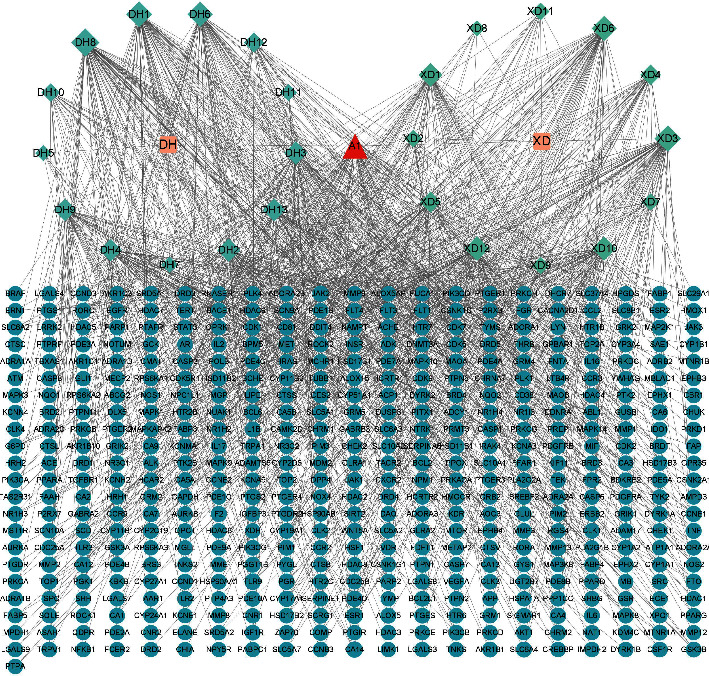
The compound-target network for DXHP. Ultramarine circles represent genes, greenish blue circles represent active compounds of DXHP, orange circles represent natural medicines of DXHP, and the red circle represents the common compounds of the DXHP.

We obtained 106 active components of DH and 63 active components of XD. After screening by OB and DL standards, 20 active ingredients with good ADME properties were obtained for the subsequent study. In addition to this, DH and XD were found to contain some pharmacologically active ingredients, which were excluded because their OB and DL values were less than the screening criteria. Thus, according to previous literature reports, we included a total of six active ingredients such as osthole, columbianadin, umbelliferone, asperosaponin VI, ursolic acid, and loganin [39, 40]. A total of 26 DXHP active compounds and their corresponding 463 targets were screened (Table 1). The results show that a single compound can regulate multiple targets, indicating that DXHP has multicomponent and multitarget components. The active compounds of DXHP and corresponding genes are shown in Figure 2.

### 3.2. Drug-Disease Core Target Acquisition

**Figure 3:**
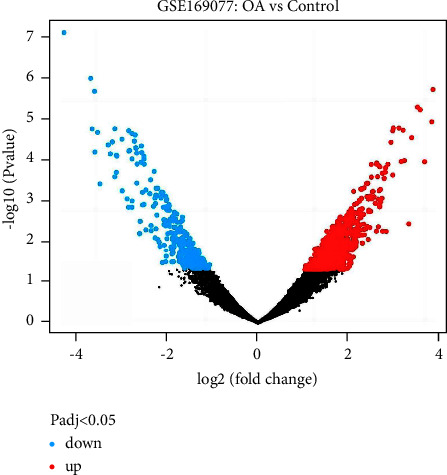
The volcano map of differentially expressed genes associated with osteoarthritis. Blue represents decrease and red represents increase.

**Figure 4:**
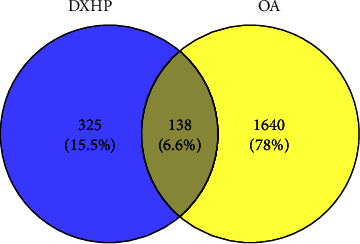
The Venn diagram showing 1778 OA-related targets and 463 DXHP-related targets. The intersection section indicates the 138 targets of DXHP in the treatment of OA.

In the GEO database, we identified the dataset that met the criteria as GSE169077. The chip contained six osteoarthritis cartilage tissue samples as the experimental group and five normal knee cartilage tissue samples as the control group. The results of differential expression gene analysis of osteoarthritis showed that 792 genes were found to be differentially expressed. The heatmap and Limma package in the R language were used to draw the dataset volcano map of differential genes (Figure 3). The GeneCards database yielded 3647 OA-related genes in total, and 885 OA-related genes were obtained using a relevance score >1 as the screening criterion. The TTD database attained 33 OA-related genes in total, whereas the OMIM database attained 30 OA-related genes. The above gene sets were combined, and duplicate values were removed to obtain a total of 1778 OA-related genes. The screened drug targets and OA-related genes were input into Venny 2.1.0 plotter, a Venn figure was constructed, and a total of 138 common targets were obtained (Figure 4).

### 3.3. PPI Network Construction and Centiscape Analysis

**Figure 5:**
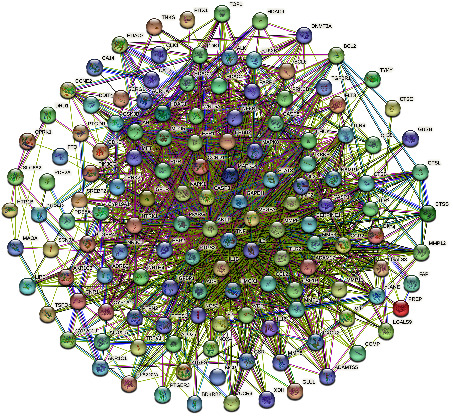
The PPI network of common targets of DXHP and OA.

**Figure 6:**
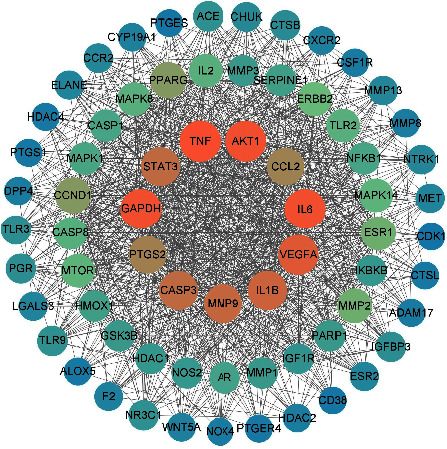
Overlapping targets of DH and XD acting on OA.

The herb-disease common targets were imported into the STRING Bioinformatics system. Species option was selected as “*Homo sapiens*,” the PPI network was constructed (Figure 5), and the CSV file was entered into Cytoscape software 3.9.1 for network topology analysis using the network Stats tool and the centiscape plugin for the construction of the three parameter correlations such as DC, BC, and CC. 138 nodes and 1599 edges made up the network, which had an average connectedness of 23.70. A total of 25 core targets were selected through the centiscape plugin by selecting genes with greater than average DC, BC, and CC parameters as key targets and ranking them by the degree centrality value. The 10 highest ranked core targets are listed in Table 2. DH and XD have 155 overlapping targets, 69 of which are associated with OA (Figure 6).

### 3.4. GO Functional Annotation

**Figure 7:**
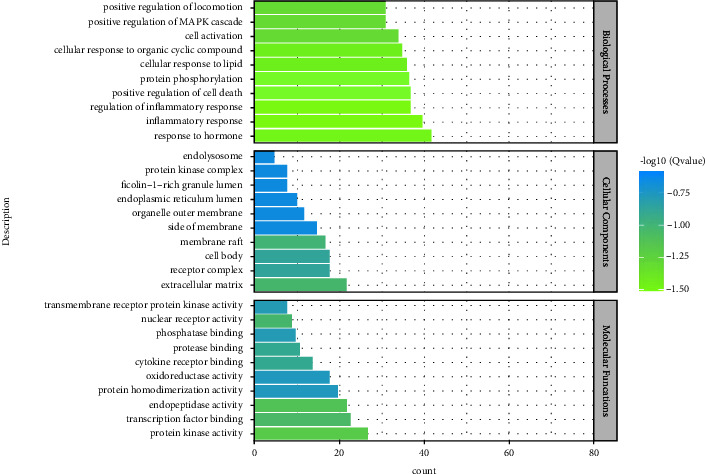
BP, CC, and MF of GO enrichment analysis.

GO functional annotation analysis showed that the enrichment results included 1705 biological processes (BPs), 155 molecular functions (MFs), and 89 cellular components (CCs). Most GO annotations of BP were contained to regulation of inflammatory response, response to hormone, inflammatory response, positive regulation of locomotion, cellular response to lipid, cellular response to organic cyclic compound, positive regulation of cell death, protein phosphorylation, positive regulation of MAPK cascade, and cell activation. MF annotation was mainly involved in protein kinase activity, endopeptidase activity, transcription factor binding, nuclear receptor activity, cytokine receptor binding, protease binding, protein homodimerization activity, transmembrane receptor protein kinase activity, oxidoreductase activity, and phosphatase binding. The majority of CC annotations were included to the extracellular matrix, membrane raft, receptor complex, cell body, organelle outer membrane, ficolin-1-rich granule lumen, endolysosome, protein kinase complex, side of the membrane, and endoplasmic reticulum lumen. Based on their *Q*-value (the *Q*-value was used for multiple testing, it was calculated using the Benjamini–Hochberg procedure, and higher *Q*-values showed the greater GO term enrichment), the top 10 terms of BP, CC, and MF were rated. The terms are presented in Figure 7.

### 3.5. KEGG Pathway Analysis

**Figure 8:**
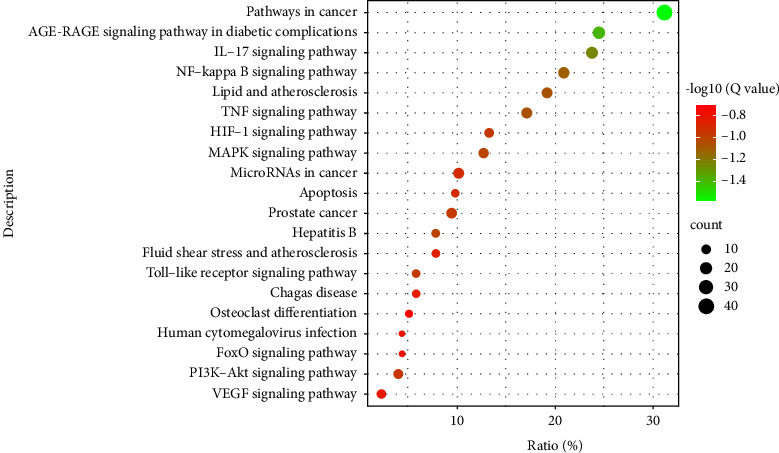
The top 20 enriched KEGG pathway.

**Figure 9:**
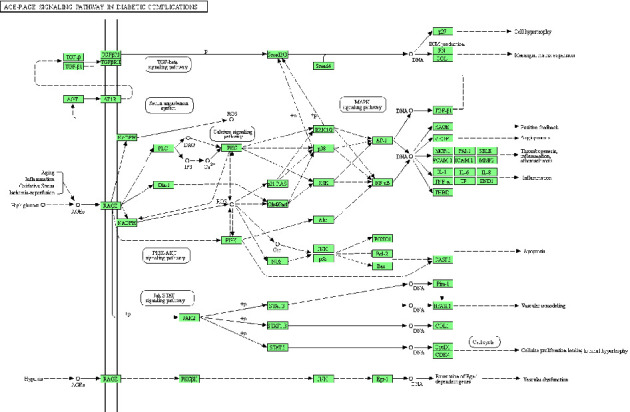
The AGE-RAGE signaling pathway. The green squares represent the core targets of this study.

**Figure 10:**
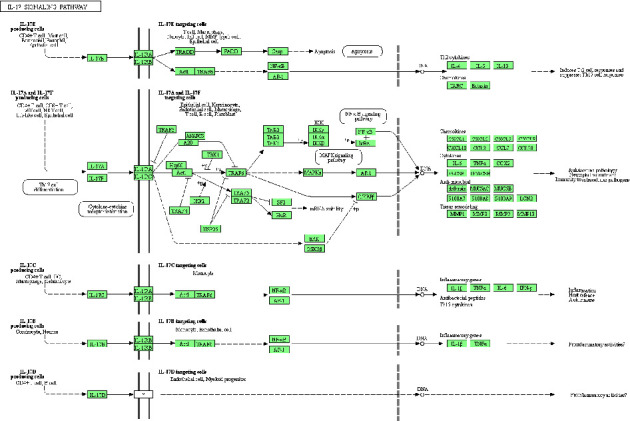
The IL-17 signaling pathway. The green squares represent the core targets of this study.

KEGG pathway analysis enriched 179 signaling pathways after FDR correction. The top 20 pathways included pathways in cancer, the AGE-RAGE signaling pathway in diabetic complications, the IL-17 signaling pathway, the NF-kappa B signaling pathway, lipid and atherosclerosis, the TNF signaling pathway, the MAPK signaling pathway, hepatitis B, the toll-like receptor signaling pathway, prostate cancer, the HIF-1 signaling pathway, the PI3K-Akt signaling pathway, microRNAs in cancer, apoptosis, fluid shear stress and atherosclerosis, chagas disease, the VEGF signaling pathway, human cytomegalovirus infection, the FoxO signaling pathway, and osteoclast differentiation. Based on their *Q*-value, the top 20 critical signaling pathways are presented in Figure 8. The figures of the two inflammation-related signaling pathways with the highest number of core genes enriched are shown in Figures 9 and 10.

### 3.6. PPI Enrichment MCODE Analysis

**Figure 11:**
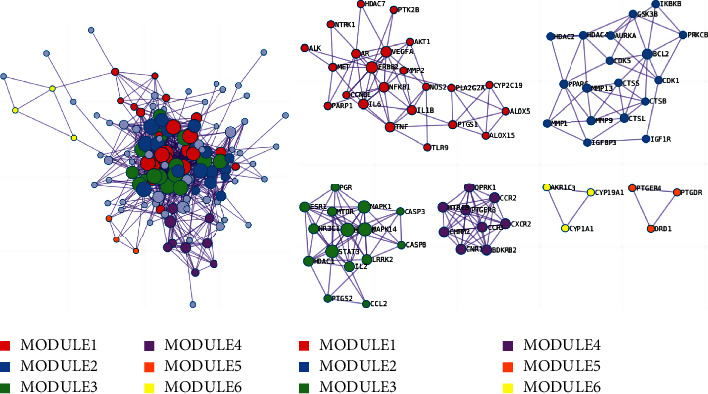
The top 6 gene clusters in the enrichment MCODE analysis.

Core genes were identified through network construction and MCODE analysis using the complete datasets for the independent enrichment analysis of gene clusters. The enrichment analysis of biological processes was used for each MCODE component (Figure 11 and Table 3). MCODE analysis showed that the core genes of AKT1, IL1B, TLR9, VEGFA, MMP2, PTGS1, NOS2, IL6, etc., are mainly involved in the biological process of regulating the inflammatory response in OA. The core genes of ESR1, MTOR, MAPK1, CASP3, CCL2, PGR, etc., are mainly involved in the biological process of regulating defense response in OA. The three terms with the highest scores based on the *P* values were used as the functional description of the corresponding components (Table 4).

### 3.7. Molecular Docking Verification

**Figure 12:**
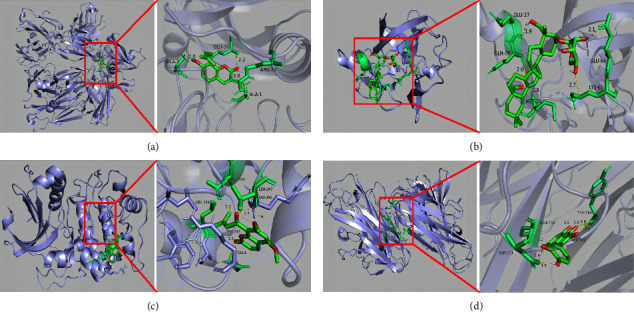
The model of molecular docking simulation results. (a) VEGFA and osthole. (b) IL1B and cauloside A. (c) AKT1 and angelicone. (d) TNF and beta-sitosterol.

To further validate the reliability of the binding of key targets and components screened by the network analysis, molecular docking verification of some key targets and important active components was carried out using Autodock Vina1.2.0 software. It is largely accepted that the steadier the binding structure, the smaller the binding energy of the receptor-ligand docking and therefore the higher the likelihood of interaction between the two, with a binding energy < −5.0 kcal/mol as the screening criterion. Binding energies < −5.0 kcal/mol indicate potential activity, and docking with binding energies < −7.0 kcal/mol is extremely stable [41]. The top six active ingredients in the component-target network were selected for molecular docking with the top six targets in the PPI network. The results showed that about 83% of targets and active components exhibited binding ability and 16% exhibited extremely strong binding ability. These molecular docking findings align with earlier network screening conclusions, which indirectly validate the treatment ability of DXHP on OA and demonstrate the reliability of network pharmacology applied to this study. The docking results of binding affinity and detailed compound-target interactions are presented in Table 5 and Figure 12.

## 4. Discussion

OA has a high prevalence in the elderly population, and modern medical treatment options are primarily symptomatic, with no options available to curb disease progression [42]. The advantages of DXHP in relieving OA symptoms as well as delaying OA disease progression have been demonstrated in previous studies [43], but its mechanism of action remains incompletely elucidated. Therefore, we investigated the mechanism of action of DSHP on OA in a more systematic way using a network pharmacology and the molecular docking approach.

According to this investigation, DXHP's primary therapeutic ingredients for OA include substances such as osthole, asperosaponin VI, angelicone, beta-sitosterol, ammidin, and cauloside A, among which *β*-sitosterol is the overlapping component of DH and XD. In recent years, the pharmacological effects of asperosaponin VI in anti-inflammatory analgesia, prevention of osteoporosis, neuroprotection, and anti-apoptosis have attracted the attention of many scholars [44]. Many in vivo and cell experiments have shown that asperosaponin VI can phosphorylate ERK/2 protein to promote the expression of osteogenic genes such as ALP, OCN, COL1 and RUNX2, which is closely related to the PI3K/Akt signaling pathway and the MAPK signaling pathway [45]. In addition, asperosaponin VI could significantly reduce MDA, TNF-*α*, IL6, and IL10 by significantly inhibiting oxidative stress and inflammatory response in tissues [46]. Osthole, one of the main active components of DH, is thought to improve bone metabolism and promote osteoblast activation [47]. Recent studies on the anti-inflammatory mechanism of osthole have found that osthole can inhibit the formation and resorption activity of osteoclasts by inhibiting the activation of NF-*κ*B and NFATc1 and reducing the expression of osteoclast-specific genes such as CTSK, MMP-9, TRAP, integrin *β*3, C-SRC, and NFATc1 [48]. *β*-sitosterol has a strong down-regulate effect on proinflammatory factors, such as IL-1*β*, IL-6, and TNF-*α* and quenches ROS produced by the human body through antioxidant activity. It can also alleviate inflammation through eosinophil percolation [49]. Chen et al. assessed the knee joints of OA rabbits by morphological and histological methods and found that *β*-sitosterol could significantly inhibit the secretion of matrix metalloproteinases (MMPs) and inhibit the degradation of cartilage [50].

The observations of the coincide target PPI analysis demonstrated that the core targets of DXHP in the healing of OA encompassed multiple targets such as TNF, GAPDH, IL6, AKT1, IL1B, VEGFA, CASP3, STAT3, MMP9, and PTGS2. TNF-*α* has been demonstrated in many diseases, including osteoarthritis, autoimmune diseases, ankylosing spondylitis, insulin resistance, psoriasis, nephropathy, and cancer [51]. TNF-*α* is associated with many cytokines, and it has been found that TNF-*α* implicated in angiogenesis by synergistic induction of VEGF production with IL-1*β* and IL-6 [52]. Nie et al. found that TNF-*α* could regulate the harmony between Treg cells and TH17 and TH1 in the joint synovium through FOXP3 dephosphorylation [51]. When cells are stimulated with NO, GAPDH will be nitrosylated, bound to E3 ubiquitin ligase Siah1, and undergo nuclear translocation and apoptosis [53]. The results of molecular docking suggest that DXHP may achieve the purpose of treating OA by binding to GAPDH. Studies have shown that [54] knockdown of the IL-6 gene in an OA rat model can lead to inhibition of MMP13 expression and secretion, which may be related to the inhibition of c-fos/ap-1-mediated inflammatory stimulation in OA chondrocytes. IL-6 can also cause an increase in the expression of MMP9, prevents the formation of type II collagen and proteoglycans, accelerates the degradation of extracellular matrix, and influences bone resorption by activating osteoclasts [55]. Shahine and Elhadidi found that the expression amount of the IL-1*β* gene in OA organisms was positively correlated with the pain index [56]. Correspondingly, high expression of IL-1*β* was detected in the synovial membrane and fluid of OA organisms and was positively correlated with OA disease [57]. Apoptotic chondrocytes are essential for the progression of OA. AKT1 is a key down-regulate gene kinase involved in the PI3K pathway. Researchers have demonstrated that the PI3K/AKT pathway that involves AKT1 prevents chondrocyte apoptosis [58]. TP53 inhibits DNA replication, induces apoptosis, and accelerates cartilage degradation [59]. In addition, researchers have demonstrated that the VEGFA is closely associated with many pathological responses, such as osteophyte formation and cartilage degeneration in OA [60]. The expression of VEGFA stimulates the division of vascular endothelial cells to promote angiogenesis, accelerate the exchange of nutrients in the knee joint and the metabolism of inflammatory products, and promote the growth of cartilage synovial cells.

The KEGG enrichment analysis showed that the main enriched inflammation-related signaling pathways included AGE-RAGE, IL-17, NF-kappa B, MAPK, TLR, HIF-1, PI3K-Akt, and the VEGF signaling pathway. The other signaling pathways enriched included pathways in cancer, lipid and atherosclerosis, hepatitis B, prostate cancer, microRNAs in cancer, apoptosis, fluid shear stress and atherosclerosis, chagas disease, human cytomegalovirus infection, FoxO signaling pathway, and osteoclast differentiation. The MCODE analysis revealed that the regulatory response to inflammation was the most significant biological process in treating OA by DXHP. The NF-*κ*b pathway is closely related to cartilage destruction in OA and targeted therapy of OA [61]. Previous research has showed that the secretion level of NF-*κ*b in joint synovial fluid and peripheral blood of OA patients is increased, and the degree of up-regulation of the NF-*κ*b pathway is positively correlated with the degree of cartilage erosion and destruction [62]. After phosphorylation, I*κ*B*α* dissociates from NF-*κ*B, and NF-*κ*B is activated and enters the nucleus, promoting the synthesis and secretion of TNF-*α* and IL-6 [63, 64] and eventually leading to the degeneration of articular cartilage [65, 66]. In addition, IL-17 can trigger the release of chemokines, cytokines, antimicrobial peptides, and matrix metalloproteinases from mesenchymal and bone marrow cells [67]. Bai et al. [68] found that the level of IL-17 in the serum of OA patients increased and was positively correlated with the severity of OA. Due to the lack of capillaries in articular cartilage, the cartilage microenvironment is essentially a hypoxic environment. The main hypoxia-inducible factor (HIF) of articular chondrocytes is a transcription factor, which is also the main mediator of homeostatic response that enables cells to survive under hypoxic conditions [69]. In OA, the expression of HIF-1*α* decreases [70], and the loss of HIF-1*α* can up-regulate the expression of MMP13, degrade col2A1 and ACAN, promote chondrocyte degradation, and promote the development of OA [71]. The advanced glycation end product receptor (RAGE) is secreted in a variety of cells, including macrophages and mast cells [72]. AGE is a ligand of RAGE and is the end product of glycosylation of proteins and sugar [73]. RAGE is essential for the induction of several inflammatory genes as well as important signaling pathways linked to proinflammatory responses. Although AGE induces inflammation by exciting NF-*κ*B and MAPK in a variety of cells including osteocytes [74], it can also up-regulate the expression of PGE2 and NO via the MAPK pathway and induces the chondrocytes inflammatory [75].

As an herb pair, DH and XD have many overlapping targets and active ingredients, and the compatibility mechanism of DXHP in treating OA may be related to this. *β*-sitosterol, as its overlapping active ingredient, is associated with anti-inflammatory and antioxidant effects, immune regulation, and bone metabolic balance [76]. Furthermore, the aforementioned KEGG and MCODE analyses revealed that the overlapping targets such as IL6, IL-1*β*, AKT1, VEGFA, MMP13, and STAT3 are vital links in inflammatory signalling pathways such as AGE-RAGE, IL-17, MAPK, and NF-kappa B and are extensively involved in the biological processes of regulating the inflammatory response in OA. This finding may help us to better understand the compatibility mechanism of DXHP from a molecular perspective.

Molecular docking tests were carried to further confirm the molecular mechanism of DXHP in the treatment of OA. The results revealed that osthole, angelicone, cauloside A, and *β*-sitosterol have excellent binding activity with multiple key targets, such as TNF, GAPDH, IL-6, AKT1, IL-1*β*, and VEGFA. This also explains the therapeutic mechanism of DXHP from another perspective. These results offer insight for the subsequent application of network pharmacology methods to improve the efficiency of natural medicine ingredient development, which could facilitate the development of new high-efficiency, low-toxicity, multi-target OA drugs capable of improving symptoms and delaying disease progression.

Compared to recently published network pharmacology studies of similar diseases [77], we have expanded the source of the drug and disease database and introduced differential genes from the GEO microarray, making the source more comprehensive. In order to capture modules with high-quality biological processes in large PPI networks, we apply the MCODE clustering algorithm, which allows for a further in-depth interpretation of the target network. In addition, we have investigated the overlapping active ingredients and targets of this herb pair to elucidate the compatibility mechanisms of DXHP from a new perspective.

Despite the study's advantages, several limitations should be mentioned. Firstly, the sources of component and target data are scattered and vary widely between databases, resulting in insufficiently accurate data sources [78]. Secondly, the validation of the findings is only based on computer simulations of molecular docking, which has limitations in terms of convincingness [79]. Therefore, the key pathways and targets identified in this study need to be validated in more in-depth in vivo and in vitro experiments.

## 5. Conclusion

In this study, we found that DXHP is a valuable TCM herb pair for treating patients with OA through multiple components, targets, and pathways. Its pharmacological mechanism could be through the AGE-RAGE, IL-17, NF-kappa B, MAPK, TLR, HIF-1, PI3K-Akt, and VEGF signaling pathways to alleviate OA. Hopefully, our research may provide a scientific basis for the prodrug discovery of its natural ingredients and the identification of therapeutic targets in the future.

## Figures and Tables

**Table 1:** Active compounds of DXHP.

Drug	Serial no.	MOL ID	Active compound	OB	DL
DH	DH1	MOL001941	Ammidin	34.55	0.22
	DH2	MOL001942	Isoimperatorin	45.46	0.23
	A1	MOL000358	Beta-sitosterol	36.91	0.75
	DH3	MOL003608	O-acetylcolumbianetin	60.04	0.26
	DH4	MOL004777	Angelol D	34.85	0.34
	DH5	MOL004778	[(1R,2R)-2,3-dihydroxy-1-(7-methoxy-2-oxochromen-6-yl)-3-methylbutyl] (Z)-2-methylbut-2-enoate	46.03	0.34
	DH6	MOL004780	Angelicone	30.99	0.19
	DH7	MOL004792	Nodakenin	57.12	0.69
	DH8	MOL000614	Osthole	38.75	0.13
	DH9	MOL002905	Columbianadin	14.82	0.36
	DH10	MOL001950	Psoralen	33.06	“0.10
	DH11	MOL003621	Meranzin hydrate	43.61	0.17
	DH12	MOL005785	Bergaptol	24.22	0.12
	DH13	MOL002558	Umbelliferone	27.37	0.05

XD	XD1	MOL003152	Gentisin	64.06	0.21
	A1	MOL000358	Beta-sitosterol	36.91	0.75
	XD2	MOL009312	(E,E)-3,5-di-O-caffeoylquinic acid	48.14	0.68
	XD3	MOL009316	Cauloside A	43.32	0.81
	XD4	MOL008188	Japonine	44.11	0.25
	XD5	MOL009323	Sylvestroside III	48.02	0.53
	XD6	MOL003106	Asperosaponin VI	1.67	0.07
	XD7	MOL000651	Sweroside aglycone	68.68	0.08
	XD8	MOL000652	Venoterpine	68.97	0.04
	XD9	MOL009313	Mesitol	65.67	0.03
	XD10	MOL000511	Ursolic acid	16.77	0.75
	XD11	MOL001680	Loganin	5.90	0.44
	XD12	MOL000296	Hederagenin	36.91	0.75

**Table 2:** Top 10 core targets of PPI analysis.

Targets	DC	BC	CC
TNF	96	0.076310133	0.740540541
GAPDH	95	0.088430017	0.740540541
IL6	91	0.05922158	0.721052632
AKT1	90	0.069715376	0.724867725
IL1B	85	0.052242761	0.698979592
VEGFA	82	0.067869259	0.695431472
CASP3	75	0.042146765	0.671568627
STAT3	70	0.024000163	0.643192488
MMP9	69	0.028131984	0.637209302
PTGS2	68	0.028605206	0.634259259

**Table 3:** Biological processes of protein-protein interaction in the enrichment.

Module	GO	Description	Log10 (*P*)
MODULE1	GO: 0043410	Positive regulation of MAPK cascade	−15.4
	GO: 0043408	Regulation of MAPK cascade	−13.7
	GO: 0033674	Positive regulation of kinase activity	−13.6

MODULE2	GO: 0022411	Cellular component disassembly	−13.3
	GO: 0030574	Collagen catabolic process	−13.1
	GO: 0032963	Collagen metabolic process	−12.0

MODULE3	GO: 0071407	Cellular response to organic cyclic compound	−12.3
	GO: 0032496	Response to lipopolysaccharide	−10.0
	GO: 0002237	Response to molecule of bacterial origin	−9.8

MODULE4	GO: 0007204	Positive regulation of cytosolic calcium ion concentration	−11.2
	GO: 0007188	Adenylate cyclase-modulating G protein-coupled receptor signaling pathway	−10.8
	GO: 0051048	Negative regulation of secretion	−9.1

MODULE5	GO: 0007204	Positive regulation of cytosolic calcium ion concentration	−6.5

MODULE6	GO: 0006694	Steroid biosynthetic process	−7.3
	GO: 0034754	Cellular hormone metabolic process	−7.1
	GO: 0120254	Olefinic compound metabolic process	−6.9

**Table 4:** The top three biological processes in enrichment MCODE analysis.

GO	Description	Log10 (*P*)
GO: 0050727	Regulation of inflammatory response	−37.1
GO: 0006954	Inflammatory response	−35.7
GO: 0031347	Regulation of defense response	−35.7

**Table 5:** The binding ability of active compounds to core targets.

Object	Binding energy (kcal/mol)						
Target	PDB ID	DH8	XD3	DH1	DH6	A1	XD6
TNF	2AZ5	−6.63	−6.67	−5.62	−6.52	−9.55	−5.01
GAPDH	1IHY	−5.82	−6.81	−5.58	−6.35	−5.76	−3.16
IL6	1ALU	−5.47	−6.73	−5.84	−6.41	−8.03	−4.13
AKT1	3MV5	−5.61	−6.51	−6.03	−6.69	−7.79	−3.12
IL1B	6Y8M	−6.07	−8.01	−4.93	−6.26	−7.98	−3.91
VEGFA	6T9D	−6.68	−5.99	−5.92	−6.05	−8.06	−2.29

## Data Availability

The original contributions presented in the study are included within the article/supplementary material. The data supporting the findings of the current study are available from the corresponding authors upon request.
